# Skin color reporting in basal cell carcinoma-related randomized controlled trials in top dermatology journals: a systematic review

**DOI:** 10.1007/s00403-024-03187-7

**Published:** 2024-07-05

**Authors:** Natasha L. Salmen, Deven P. Curtis, Anthony N. Baumann, Jared Willets, Robert T. Brodell

**Affiliations:** 1 College of Medicine, Northeast Ohio Medical University, 5150 Lower Elkton Rd. Leetonia, Rootstown, OH 44431 USA; 2 University of Mississippi Medical Center, Mississippi and JV “Sonny” Montgomery Veterans Hospital, Jackson, MS USA

**Keywords:** Basal cell carcinoma, Skin of color, Fitzpatrick scale, Race

## Abstract

Objectives: To determine the rate skin color is reported in randomized controlled trials (RCTs) involving basal cell carcinoma (BCC) identification and treatment in the top ten dermatology journals. Methods: A systematic review was conducted of RCTs involving BCC among the top ten dermatology journals, determined by impact factor, from inception to July 11th, 2023. Studies were included if they reviewed the prevention, detection, and treatment of BCC, directly involved patients, and were classified as RCTs. Studies were classified as positive for reporting skin of color (SOC) if the demographic data in the methods or results included any of the following terms: Fitzpatrick scale, race, ethnicity, skin of color, or sunburn tendency. Results: Of the 51 studies identified, only 23 articles reported data pertaining to skin color within the results section (45.1%); whereas 28 articles mentioned skin color somewhere within the text (54.9%). Subgroup analysis was performed, and no statistical significance was found for study location or year of publication. Conclusion: Dark skin color can make it more difficult to diagnose skin tumors and it is unknown if race affects response to treatment. Less than 50% of RCTs related to basal cell carcinoma in top international dermatology journals included skin color within the demographic portion of their results section pertaining to study participants. Subgroup analysis demonstrated that studies performed within the United States reported skin color less than half the time (40%). Additionally, there has been no statistically significant difference in reporting over the past 4 decades. Further research is necessary to determine whether low reporting rates of race/skin color in BCC-related RCTS could impact diagnostic or treatment recommendations for patient care in this group.

## Introduction

Basal cell carcinoma (BCC) is the most common type of skin cancer, as well as the most common malignancy of all cancers [1]. 3.6 million cases of BCC are reported each year in the United States, which correlates with one in every five Americans over their lifetimes [2, 3]. The incidence rate for BCC worldwide has increased over the last 30 years. Although BCC is not commonly associated with mortality, it can be damaging to local tissue [3]. Thus, early detection and prompt treatment are important.

BCC arises from the basal layer of epidermis from stem cells located within the interfollicular epidermis [4]. Some nonmodifiable risk factors for developing BCC include age, gender (male/female ratio is 2:1), fair skin, and genetic predisposition. The most important risk factor, as well as a modifiable risk factor, includes ultraviolet (UV) sun exposure. Most BCC lesions occur on areas of the body most susceptible to sun damage [5].

Although BCC is more common in patients with lighter skin tones, this cancer can also present in patients with skin of color (SOC). BCC is the second most common skin cancer in black patients, and accounts for 20–30% of skin cancers in this group [5]. It is also the most common skin cancer in Hispanic, Chinese, and Japanese populations [6]. The clinical presentation in these patients is altered in patients with darker skin coloration. Telangiectasias and erythema are disguised by darker skin coloration. In addition, more than 50% have BCC with a pigmented appearance in patients with SOC compared to only 5% in white patients. Nodular basal cell carcinoma can be confused with nodular melanoma in this circumstance [6]. The decreased frequency of BCC may be due to the UV protective effect of melanin pigment which can produce a sun protection factor (SPF) of 2 or 3 in some patients. Patients with black skin also absorb UV heating the skin when compared to white skin which reflects UV. This may cause patients with darker skin to seek shade or cover up more effectively.

To better understand BCC in SOC, it is essential studies include participants of all races and skin tones. The number of randomized controlled trials (RCTs) involving BCC reporting skin color has not been previously reported. The purpose of this systematic review is to find out the rate of race, ethnicity, or skin color reporting in RCTs involving BCC in the top ten most impactful dermatology journals worldwide.

## Methods

### Study design

**Fig. 1:**
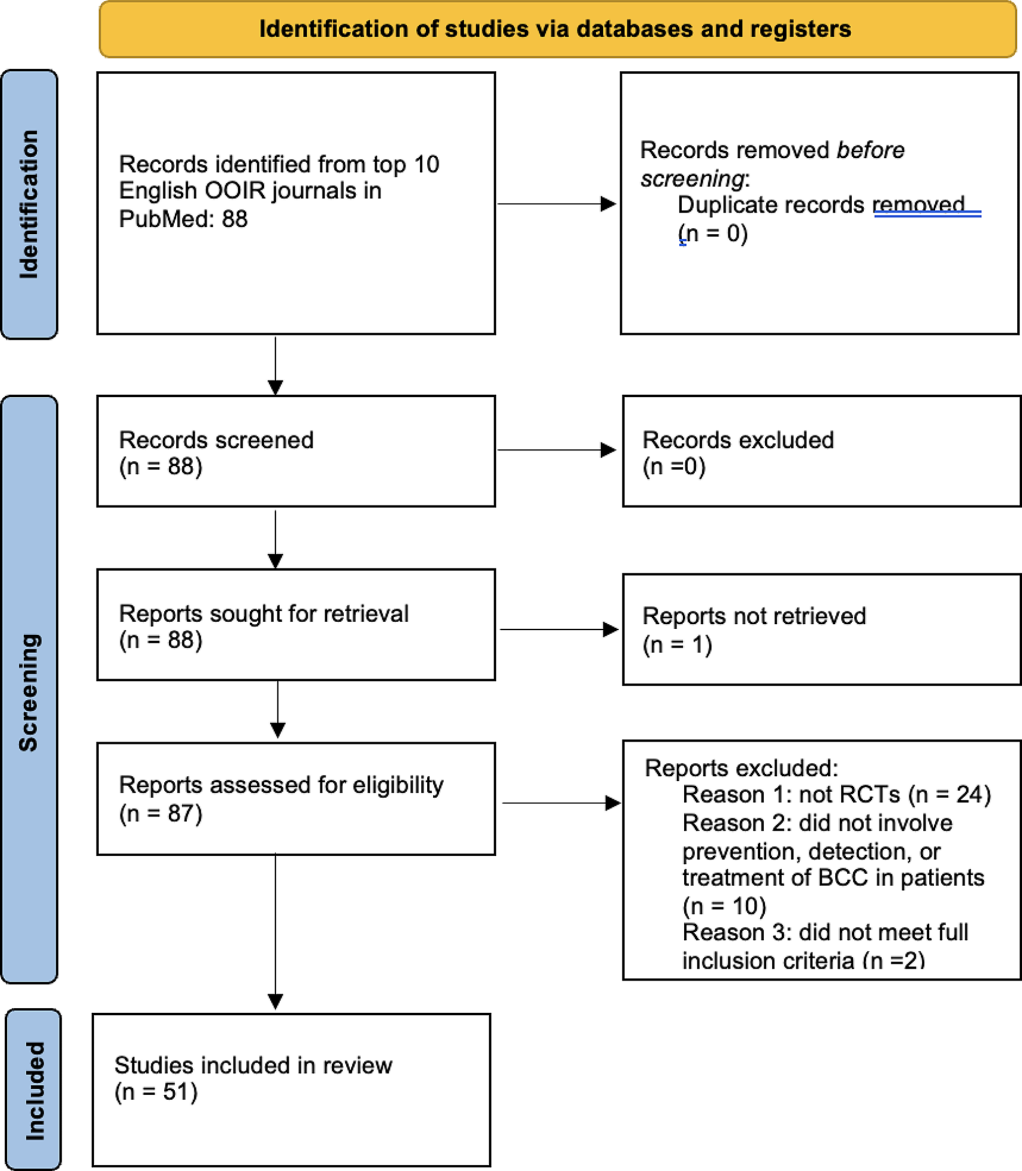
Preferred Reporting Items for Systematic Review and Meta-Analysis (PRISMA) diagram

A systematic review of RCTs involving BCC was conducted from inception to July 11, 2023. The top ten most impactful dermatology journals, written in English, in the world were included. The included journals were chosen from the Observatory of International Research rank list (see Table 1). The journal ranked 9th was the Journal der Deutschen Dermatologischen Gesellschaft, which was written in German. Therefore, this journal was excluded based on our inclusion criteria, and the 11th journal, Dermatitis, was selected instead. The database PubMed was utilized to conduct the search. The search terms included “basal cell carcinoma” and the abbreviations of the ten journals, which can be found in Table 1. The filter RCT was also applied. This study follows the Preferred Reporting Items for Systematic Review and Meta- Analyses (PRISMA), which can be seen in Fig. 1.

**Table 1:** The top ten most impactful dermatology journals, determined by impact factor, from the Observatory of International Research, July 2023

Journal:	Impact Factor:	PubMed Term:
Journal of the American Academy of Dermatology	15.49	J Am Acad Dermatol
JAMA Dermatology	11.82	JAMA Dermatology
British Journal of Dermatology	11.11	Br J Dermatol
Journal of the European Academy of Dermatology and Venerology	9.23	J Eur Acad Dermatol Venereol
Journal of Investigative Dermatology	7.59	J Invest Dermatol
Contact Dermatitis	6.42	Contact Dermatitis
American Journal of Clinical Dermatology	6.23	Am J Clin Dermatol
Journal of Dermatological Science	5.41	J Dermatol Sci
Dermatology	5.2	Dermatology
Dermatitis	5.19	Dermatitis

### Inclusion and exclusion criteria

Inclusion criteria included: RCT articles that related to BCC prevention, detection or treatment, involved patients, and were written in English. Exclusion criteria included: if they were not RCT, did not involve patients (such as educational objectives or cost effectiveness of treatments), or if the full text was not available.

### Study categories

A subgroup analysis was performed on the following categories: study location (conducted within the United States versus outside of the United States) and year (prior to 2005, 2005–2015, and 2016 and after).

### Primary outcome measures

The primary outcome was to report any mention of a term indicating skin color. Some of these terms include ethnicity, race, Fitzpatrick rating, skin tone, skin color, and phototype. The primary outcome measure was stratified by study location and year of publication. Articles that indicated skin color were evaluated for the race and/or skin color type that was reported for study participants.

### Article sorting process

The articles initially collected were sorted in Rayyan.ai, which is an online software designed to organize articles for systematic reviews. The articles were screened and categorized. The author DC removed duplicate articles in this software if any existed.

### Data extraction

Articles were imported into a Google Sheets spreadsheet. Three of the coauthors (NS, DC, and JW) screened the articles for inclusion criteria. The extracted data was organized into the following:

- Journal
- Title
- Authors
- Years
- Full text availability
- Study type
- Involves treatment, prevention, detection of BCC in patients
- Include or exclude
- Reason for exclusion
- Mentions skin color/race/ethnicity/skin type
- Reports skin color/race/ethnicity/skin type in patient demographics
- Fitzpatrick type included
- Terms used to describe skin
- Funding source
- Study location

### Statistical analysis

The Statistical Package for the Social Sciences (SPSS) version 29.0 (Armonk, NY: IBM Corp) was used in this study for statistical analysis. Fisher’s exact test was utilized for comparison between categorical groups. All p-values (2-sided) less than 0.05 were considered statistically significant.

## Results

### Initial search results

A total of 88 articles were initially retrieved with one article being excluded for lack of full-text, leaving 87 articles evaluated by full-text screening. Twenty-four articles were then excluded for not being a randomized controlled trial, leaving 63 randomized controlled trials. Ten articles were then excluded for being unrelated to the treatment, prevention, or detection of BCC in humans, leaving 53 randomized controlled trials. Two other articles were then excluded for not meeting full inclusion criteria. At the end of article sorting, 51 randomized controlled trials were included in this systematic review (Fig. 1 below).

### Reporting demographics

From the 51 included randomized controlled trials, 23 articles (45.1%) reported on race and/or skin color in the results section of the manuscript whereas 28 articles (54.9%) mentioned race and/or skin color anywhere in the manuscript. Additionally, a total of 20 articles (39.2%) reported the Fitzpatrick scale indicating that of the 23 articles that reported on race and/or skin color, the Fitzpatrick scale was used in 87.0% (*n* = 20) of those articles when reporting on race and/or skin color was present. Other than the Fitzpatrick scale, other types of race and/or skin color mentioned or reported were white/Caucasian (*n* = 7 counts), American Indian (*n* = 1 count), Native Hawaiian/Pacific Islander (*n* = 1), and other (*n* = 2 counts).

### Reporting rate by date

Seven articles (50%) of the 14 articles prior to 2005, 6 articles (37.5%) of the 16 articles from 2005 to 2015, and 10 articles (47.6%) of the 21 articles from 2016 and later reported on race and/or skin color with no statistically significant difference in the rate of reporting between cohorts by year of publication (*p* = 0.755). Seven articles (50%) of the 14 articles prior to 2005, 8 articles (50%) of the 16 articles from 2005 to 2015, and 13 articles (61.9%) of the 21 articles from 2016 and later mentioned race and/or skin color with no statistically significant difference in the rate of mentioning between cohorts by year of publication (*p* = 0.702).

### Reporting rate by study location

From the 51 articles, there were 15 articles (29.4%) that were conducted in the United States and 36 articles (70.6%) that were conducted outside of the United States or in an unknown location. Six articles (40.0%) out of 15 articles conducted in the United States and 17 articles (47.2%) conducted outside of the United States or in an unknown location reported on race and/or skin color with no statistically significant difference in the rate of reporting between cohorts by study location (*p* = 0.637). Six articles (40%) out of 15 articles conducted in the United States and 22 articles (61.1%) out of 36 articles conducted outside of the United States or in an unknown location mentioned race and/or skin color with no statistically significant difference in the rate of mentioning between cohorts by study location (*p* = 0.167).

## Discussion

The goal of this paper was to systematically review the rate of reporting skin color in RCTs involving the identification, diagnosis, and treatment of BCC. Skin color was reported less than half the time in the RCTs identified (45.1%). Insufficient research regarding BCC in SOC limits the knowledge required to provide the best patient care and is similar to recent findings in RCTs related to squamous cell carcinoma [7]. Of the 23 articles reporting skin color, 20 reported utilizing the Fitzpatrick scale (87%). This scale provides a method for categorizing skin color without the need to assess for race or ethnicity. Thus, patients classified as “white” or “black” may have a range of skin tones. Not surprisingly, many of the studies in this systematic review that utilized the Fitzpatrick scale included only study participants with types that fell between I-III (lighter skin tones) since the prevalence of BCC is much higher in this group [5].

There was no significant difference in skin color reporting between years of publication comparing prior to 2005, 2005–2015, and 2016 and after. However, it appears skin color reporting in RCTs related to BCC has been increasing over the years. Prior to 2005, it was reported 50% of the time. During 2016 and after, it was reported 61.9% of the time. This increase, although not statistically significant, may highlight that studies are trying to be more inclusive and representative of all skin colors. Similarly, there was no statistically significant difference was found between studies reporting skin color within the United States (40%) compared to studies performed outside the United States (47.2%).

### Controversies related to Fitzpatrick skin color scoring system

Some authors have pointed out problems associated with the Fitzpatrick skin color rating system [8, 9]. Both patients and physicians assume someone with a higher Fitzpatrick score, indicating a darker skin tone, may be at lower risk for skin cancer. This may be incorrect. The correlation between skin pigmentation and the risk of skin cancer is not uniform [9]. While Fitzpatrick phenotypes in Caucasians correlate with skin cancer risk, the Fitzpatrick scale phototypes of non-Caucasian minorities with SOC in Thailand, Korea, and Colombia did not correlate with skin cancer risk [8–12]. One reason for this may be due to the fact that the Fitzpatrick scale uses sunburn potential as a determining factor for phototype, and many physicians underestimate the sunburn risk in non-Caucasian patients [9]. Racial and genetic differences related to handling UV injury may have more of an impact than skin pigmentation and risk of sunburn [9]. Thus, a brown African American with the same skin coloring as a brown Asian may have entirely markedly different genetic make-ups that impacts the potential for cancer. It may be important to assess both race and skin color in RCTs of basal cell carcinoma.

### Controversies related to use of race in medical records

It has been recommended that skin tone rather than race, be recorded when performing a physical examination [13]. It is argued that the inclusion of race in the history of present illness (HPI) is unnecessary for diagnosis and treatment and can lead to poor patient outcomes due to structural racism [13–17]. Some argue that racial differences seen in many diseases are due to social determinants of health rather than race, and it is the social determinants of health that should be incorporated into EHRs [18]. For research purposes and patient care, we argue, however, that race could be a signal that could highlight the importance of social determinants of health and should be reported in every HPI. In fact, incorporating race and ethnicity into EHRs can focus attention needed to decrease health disparities and enhance patient care [19].

In addition, disease-specific health disparities exist based on race and ethnicity that are not dependent on skin color [20]. These include conditions ranging from metabolic disorders to cancer [20]. For instance, there are also associations between race and drug efficacy and side effects. For example, there is a strong correlation between carbamazepine drug-induced Stevens-Johnson syndrome and the HLA-B1502 allele in some Asian populations [21]. Furthermore, race is associated with socioeconomic status, which impacts one’s health due to the environments in which patients live and work, their medical care, and their access to nutritious food [22]. Many people of the same race or ethnicity may eat similar foods, have the same beliefs and views, or access to healthcare services [16]. Thus, excluding race from medical records is problematic since this ignores disease-specific health disparities amongst different races and limits the ability to perform retrospective research or choose patients to participate in prospective trials.

### Lack of patient diversity in dermatology research

There is a lack of patient diversity in all dermatologic clinical trials, not just BCC trials. For example, a study was conducted to review RCTs between 2010 and 2015 on various dermatology lesions, such as psoriasis, acne, and vitiligo. This study found that only 11.3% of international RCTs (52 out of 626) reported on racial demographics [23]. Additionally, some dermatology journals, such as Dermatologic Surgery, restrict authors from using non-specific terms, such as ‘white’ or ‘black,’ to discuss patient skin color in articles. Nevertheless, including skin color or race can aid in scientific research and contribute to medical advancements. Underrepresentation of patients with SOC in dermatology research leads to gaps in knowledge and can impact the quality of patient care.

The National Institute of Health (NIH) is working to include study participants of various races and ethnicities in their clinical trials. In fact, recent pediatric clinical trials funded by the NIH included study participants representative of multiple races to reflect all segments of the population of the United States [24]. This requires a focus on the many barriers that deter non-white patients from participating in clinical trials. These includes mistrust and fear due to the historical issues related to participation of patients with SOC in research, such as the untreated syphilis study at Tuskegee [25] Other barriers include financial issues, lack of transportation, the stigma associated with participating in research, and health literacy [26].

## Conclusion

Skin color may impact the identification and treatment of basal cell carcinoma. This systematic review assessed BCC-related RCTs in the top ten dermatology journals worldwide. Less than 50% of these RCTs assessed the skin color of study participants. While BCC may have a low risk of metastasis and mortality, it can be destructive to local tissue and produce significant morbidity and mortality in patients of all skin colors. No statistical significance was found in subgroup analysis for studies conducted within the United States verses other countries. In addition, this study demonstrates no significant increase in skin color reporting over the past four decades. It is hoped that a consensus is building to include an assessment of skin color and race in BCC-related RCTs and to break down barriers that have limited these assessments in the past.

## Data Availability

No datasets were generated or analysed during the current study.
